# Priming persistence of HCV

**DOI:** 10.18632/oncotarget.5445

**Published:** 2015-08-31

**Authors:** Anita Schuch, Robert Thimme, Maike Hofmann

**Affiliations:** Department of Internal Medicine II, University Hospital Freiburg, Freiburg, Germany

**Keywords:** HCV, CD8^+^ T-cells, priming, T-cell failure

Acute infection with hepatitis C virus (HCV) is cleared in about 30% of cases with CD8^+^ T-cells being the main effector cells in viral elimination. Yet, about 70% of patients progress to chronic HCV infection that can result in chronic immunopathology. This immune-mediated liver diseases can progress to liver cirrhosis and ultimately to hepatocellular carcinoma.

Chronic HCV infection is characterized by an impaired virus-specific CD8^+^ T-cell response caused by several viral and host factors. However, the relative contribution of those and the exact mechanisms of CD8^+^ T-cell failure are still not fully illuminated. Viral escape and CD8^+^ T-cell exhaustion due to prolonged antigen exposure, for example, cause impaired virus-specific CD8^+^ T-cell responses and therefore contribute to HCV persistence. Exhausted HCV-specific CD8^+^ T-cells are characterized by impaired effector functions and the co-expression of inhibitory receptors such as PD-1, 2B4 and KLRG1 (Figure 1). In contrast, HCV-specific CD8^+^ T-cells that target epitopes that underwent viral escape mutations express fewer inhibitory receptors and are marked by CD127 expression [1] (Figure 1). Importantly, the identification of these distinct CD8^+^ T-cell phenotypes as markers for different mechanisms of CD8^+^ T-cell failure (viral escape versus CD8^+^ T-cell exhaustion) was based on analyses performed in patients with HCV-specific CD8^+^ T-cell populations that were detectable directly *ex vivo* by conventional peptide/MHC multimer staining. However, HCV-specific CD8^+^ T-cells are not detectable in most chronically HCV-infected patients by this method. The lack of *ex vivo* detectable HCV-specific CD8^+^ T-cells in the majority of chronically HCV-infected patients has raised the question whether this is due to technical constraints or to the absence of HCV-specific CD8^+^ T-cells in these patients.

**Figure 1:**
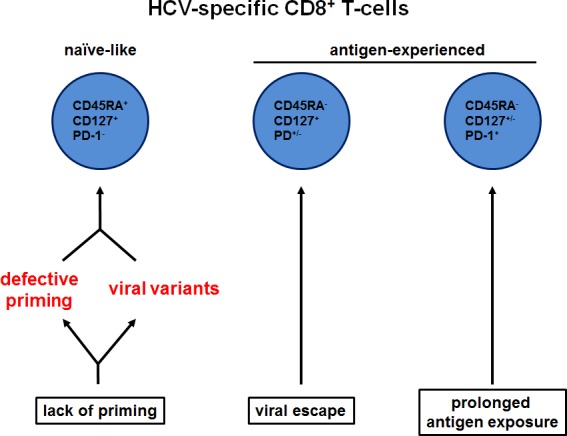
Mechanisms underlying HCV persistence reflected by CD8^+^ T-cell differentiation Beside viral escape and prolonged antigen exposure, lack of priming can cause impaired CD8+ T-cell responses and viral persistence in chronic HCV infection. These different mechanisms are reflected by diverse differentiation states of HCV-specific CD8+ T-cells.

Recently, a protocol adopted from mouse studies [2] elegantly combined peptide/MHC tetramer staining and magnetic bead enrichment to detect and analyze rare epitope-specific CD8^+^ T-cells *ex vivo* not only in infected individuals, but also in unexposed donors [3, 4]. Using this approach Nitschke *et al.* [5] were able to enrich HLA-A*02:01-restricted HCV-specific CD8^+^ T-cells specific for two different viral epitopes in seventeen analyzed patients as well as in twelve uninfected individuals. Hence, HCV-specific CD8^+^ T-cells were present in all chronically HCV-infected patients. These results indicate that the absence of virus-specific CD8^+^ T-cells does not contribute to HCV persistence in this cohort. As expected, chronically HCV-infected patients with virus-specific CD8^+^ T-cell responses that have been detectable directly *ex vivo* showed significantly higher frequencies of enriched HCV-specific CD8^+^ T-cells compared to patients without virus-specific CD8^+^ T-cell responses detectable by conventional peptide/MHC multimer staining methods (enriched detectable). Yet, enriched detectable HCV-specific CD8^+^ T-cell populations were more frequent in chronically HCV-infected patients than HCV-specific CD8^+^ T-cell precursor populations in healthy donors. A detailed phenotypic characterization of enriched HCV-specific CD8^+^ T-cells revealed that HCV-specific CD8^+^ precursor T-cells obtained from healthy donors predominantly displayed a naïve phenotype (CD45RA^+^CCR7^+^CD27^+^). In contrast, directly detectable virus-specific CD8^+^ T-cell populations in chronically HCV-infected patients had an antigen-experienced phenotype (CD45RA^−^CCR7^−^ CD27^+^). Interestingly, enriched detectable HCV-specific CD8^+^ T-cell populations had a highly heterogeneous differentiation profile with an antigen-experienced phenotype in two third and a predominantly naïve-like phenotype in one third of chronically HCV-infected patients. In addition, enriched HCV-specific CD8^+^ T-cells with an effector memory phenotype also expressed PD-1 according to previous data obtained with directly detectable HCV-specific CD8^+^ T-cells that have been defined as exhausted. Notably, the presence of both antigen-experienced and naïve-like phenotypes in different HCV-specific CD8^+^ T-cell populations within the same patients argues against a role of host genetic factors.

In a next series of experiments, the authors analyzed the functionality of enriched detectable HCV-specific CD8^+^ T-cells after co-culture with peptide-pulsed autologous monocyte-derived DC. As expected, enriched detectable HCV-specific CD8^+^ T-cells with an antigen-experienced phenotype accompanied by high PD-1 expression did not expand after co-culture, indicating CD8^+^ T-cell exhaustion. Of note, bona fide virus-specific CD8^+^ T-cell memory populations should expand upon antigen-specific stimulation. Surprisingly, only two of five naïve-like enriched detectable HCV-specific CD8^+^ T-cell populations expanded and produced effector cytokines such as IFN-γ and TNF and mobilized CD107a, a surrogate marker for degranulation. Those two patients harbored viral escape variants carrying mutations in the corresponding HCV epitope. Consequently, stimulation with the autologous variant peptide sequence did not lead to recognition and thus to successful priming of the naïve-like HCV-specific CD8^+^ T-cells in these two patients. This indicates an initial infection with a variant HCV strain, since viral escape occurring at later time points during infection would engender HCV-specific CD8^+^ T-cells with a non-naïve phenotype (Figure 1). In contrast, the three enriched detectable naïve-like HCV-specific CD8^+^ T-cell populations that did not expand upon *in vitro* priming indicate a functional impairment in priming of naïve-like HCV-specific CD8^+^ T-cells. In sum, these results indicate that in addition to viral escape and CD8^+^ T-cell exhaustion lack of priming may also represent a mechanism for HCV-specific CD8^+^ T-cell failure in chronically infected patients promoting viral persistence. However, it remains to be elucidated whether intrinsic CD8^+^ T-cell defects account for this phenomenon or whether impaired activation by DC, lack of CD4^+^ T-cell help or the presence of immunomodulatory cytokines such as IL-10 or TGF-β may play a role. It will also be important to analyze whether similar observations can be made in other chronic infections, such as chronic HBV infection, to gain further insights into the contribution of priming defects to CD8^+^ T-cell impairment and viral persistence in general.

## References

[1] Bengsch B (2010). PLoS Pathog.

[2] Obar JJ (2008). Immunity.

[3] Alanio C (2010). Blood.

[4] Schmidt J (2011). J Virol.

[5] Nitschke K (2015). J Virol.

